# Characterization of the Impact of Oncolytic Vesicular Stomatitis Virus on the Trafficking, Phenotype, and Antigen Presentation Potential of Neutrophils and Their Ability to Acquire a Non-Structural Viral Protein

**DOI:** 10.3390/ijms21176347

**Published:** 2020-09-01

**Authors:** Ashley A. Stegelmeier, Lily Chan, Yeganeh Mehrani, James J. Petrik, Sarah K. Wootton, Byram Bridle, Khalil Karimi

**Affiliations:** 1 Department of Pathobiology, Ontario Veterinary College, University of Guelph, Guelph, ON N1G 2W1, Canada; astegelm@uoguelph.ca (A.A.S.); lchan12@uoguelph.ca (L.C.); ymehrani@uoguelph.ca (Y.M.); kwootton@uoguelph.ca (S.K.W.); 2 Department of Biomedical Sciences, Ontario Veterinary College, University of Guelph, Guelph, ON N1G 2W1, Canada; jpetrik@uoguelph.ca

**Keywords:** neutrophils, oncolytic virus, vesicular stomatitis virus, trafficking, maturation, phenotype, antigen presentation, chemokine receptor, cancer, flow cytometry

## Abstract

Neutrophils are innate leukocytes that mount a rapid response to invading pathogens and sites of inflammation. Although neutrophils were traditionally considered responders to bacterial infections, recent advances have demonstrated that they are interconnected with both viral infections and cancers. One promising treatment strategy for cancers is to administer an oncolytic virus to activate the immune system and directly lyse cancerous cells. A detailed characterization of how the innate immune system responds to a viral-based therapy is paramount in identifying its systemic effects. This study analyzed how administering the rhabdovirus vesicular stomatitis virus (VSV) intravenously at 1 × 10^9^ PFU acutely influenced neutrophil populations. Bone marrow, blood, lungs, and spleen were acquired three- and 24-h after administration of VSV for analysis of neutrophils by flow cytometry. Infection with VSV caused neutrophils to rapidly egress from the bone marrow and accumulate in the lungs. A dramatic increase in immature neutrophils was observed in the lungs, as was an increase in the antigen presentation potential of these cells within the spleen. Furthermore, the potential for neutrophils to acquire viral transgene-encoded proteins was monitored using a variant of VSV that expressed enhanced green fluorescent protein (GFP). If an *in vitro* population of splenocytes were exposed to αCD3 and αCD28, a substantial proportion of the neutrophils would become GFP-positive. This suggested that the neutrophils could either acquire more virus-encoded antigens from infected splenocytes or were being directly infected. Five different dosing regimens were tested in mice, and it was determined that a single dose of VSV or two doses of VSV administered at a 24-h interval, resulted in a substantial proportion of neutrophils in the bone marrow becoming GFP-positive. This correlated with a decrease in the number of splenic neutrophils. Two doses administered at intervals longer than 24-h did not have these effects, suggesting that neutrophils became resistant to antigen uptake or direct infection with VSV beyond 24-h of activation. These findings implicated neutrophils as major contributors to oncolytic rhabdoviral therapies. They also provide several clear future directions for research and suggest that neutrophils should be carefully monitored during the development of all oncolytic virus-based treatment regimens.

## 1. Introduction

Neutrophils are a subset of granulocytes that are derived from the myeloid lineage that have well-characterized roles in clearing bacterial infections. They are also the most prevalent leukocyte in the blood and rapidly respond to perturbations caused by pathogens. Neutrophils contribute to a pro-inflammatory response and can release cytokines, such as interleukin (IL)-10, IL-12 [1], and IL-17 [2]. Recent research has demonstrated that these myeloid cells also have critical roles in clearing viral infections [3,4]. Indeed, neutrophils have been found at the sites of numerous viral infections, including influenza virus [5] and respiratory syncytial virus [6] within the lungs. In contrast, experimental models have demonstrated that they are not involved in all viral infections, such as cutaneous lesions caused by herpes simplex virus [7].

Oncolytic viruses (OVs) preferentially replicate in and destroy tumour cells while leaving most healthy cells unharmed. Importantly, oncolytic virotherapies represent promising forms of immunotherapy, and are at the cutting edge of current cancer research efforts. Vesicular stomatitis virus (VSV) is a rhabdovirus that shows promise in treating a variety of cancers [8,9]. VSV is currently being tested in several phase I clinical trials [8], and it is a promising candidate because human infections are either asymptomatic or mild, and there is a lack of pre-existing immunity within the human population that could otherwise limit its therapeutic potential. This interferon-sensitive OV has the capability to infect and lyse tumour cells because their antiviral interferon pathways are often defective. One of the distinctive features of VSV is its pan-tropism to cell surface molecules [10,11], allowing VSV-based oncolytic virotherapies to target a wide range of tumour types, but does not allow VSV to distinguish non-malignant (“normal”) cells from cancer cells.

Although VSV’s direct oncolytic potential and ability to promote tumour-specific T cell and antibody responses have been well-characterized [12,13], its effects on innate leukocytes has not been fully elucidated. A VSV-∆m51 variant was able to reach higher intratumoural titers and oncolysis when neutrophils were depleted with antibodies in a hepatocellular carcinoma model [14]. The neutrophils suppressed replication of VSV and prevented the full therapeutic potential from being obtained in this model. Within a melanoma model, the innate myeloid differentiation factor 88 (MyD88) response was determined to be an important factor in the reduction of tumour sizes mediated by VSV [15]. Although MyD88 signalling reduced VSV replication, it also reduced viral dissemination from the tumour. MyD88 is a critical protein in Toll-like receptor pathways, and *MyD88*^-/-^ knockout mice had a decrease in neutrophil infiltration into B16 melanomas that expressed the surrogate antigen chicken ovalbumin, and this correlated with reduced efficacy of the oncolytic virotherapy. One method by which VSV has an anti-tumour effect is by infecting tumour neovasculature; specifically, tumour-conditioned endothelial cells [16]. In this scenario neutrophils are critical in initiating microclots within the tumour vasculature. This phenomenon has been dubbed “acute vascular shutdown”, since it was observed within six hours of administering VSV and the effect lasted at least 24-h [16]. These results implicate roles for neutrophils during VSV-mediated oncolytic virotherapy. However, the literature in this area is limited and no studies, to date, have focused exclusively on the role of neutrophils beyond the tumour microenvironment following infection of a host with VSV.

Various models have shown that neutrophils have complex roles in oncolytic virotherapies, and whether these have positive or negative outcomes may be influenced, at least in part, by both the tumour type and the nature of the OV. Therefore, studying the impact of OVs on neutrophil responses in tumour-free hosts can provide baseline data that can subsequently be built upon by the introducing the nuances of different tumour models. Neutrophils are known to be the first responders to most infections, yet their roles in viral infections, especially those mediated by OVs is poorly studied. Therefore, we hypothesized that trafficking of this leukocyte subset dominates the acute phase of the innate immune response to VSV. Further potentially direct effects of VSV on neutrophil phenotype and functions were assessed. The results of this study demonstrate that a single dose of VSV administered intravenously led to rapid mobilization and maturation of neutrophils. Further, simulation of multi-dosing protocols that are often incorporated into oncolytic virotherapies suggested this could cause an unexpected decline in neutrophil numbers if treatment intervals are too short. As such, the findings presented here lay the groundwork for understanding how a common oncolytic rhabdovirus influences neutrophil responses in infected tumour-free hosts, the relevance of which can be extended to patients receiving oncolytic virotherapies, as well as ungulates for which VSV is pathogenic. It also has potentially important implications specifically for multi-dosing regimens with OVs.

## 2. Results

### 2.1. Neutrophils in the Bone Marrow of Mice Rapidly Responded to Infection with VSV

**Figure 1:**
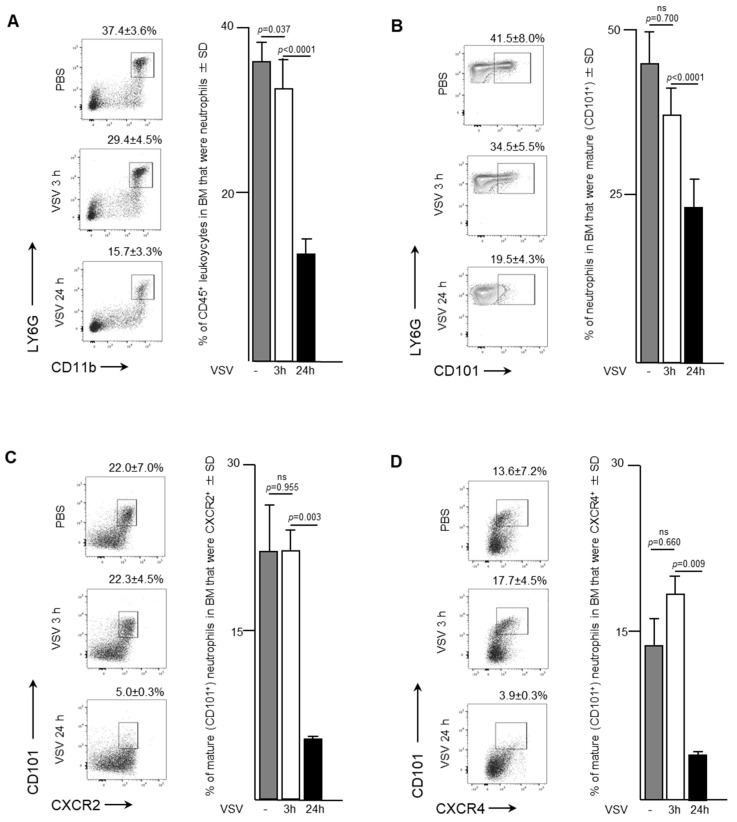
Neutrophils rapidly emigrated from bone marrow in response to infection with vesicular stomatitis virus (VSV). Six- to eight-week-old female C57BL/6 mice were injected intravenously with 1 × 10^9^ plaque-forming units of VSV or phosphate-buffed saline (PBS) and euthanized three- or 24-hours (h) later. Bone marrow flushed from femurs was used to quantify neutrophils by flow cytometry. Representative dot plots and graphs with means and standard deviations (SD) (*n* = 8/group) are shown for percentages of (**A**) CD45^+^ leukocytes that were neutrophils (defined as CD45^+^Ly6G^hi^CD11b^hi^), (**B**) neutrophils that were mature (defined by the additional expression of CD101), and (**C**,**D**) mature neutrophils expressing the lung-homing chemokine receptors (**C**) CXCR2 or (**D**) CXCR4. Data were assessed by one-way analysis of variance with Tukey’s multiple comparisons test. (ns = not significantly different).

Naïve female C57BL/6 mice received a single dose of VSV to determine the effects an oncolytic rhabdovirus had on neutrophil trafficking and phenotype over time. Samples were taken from the bone marrow of mice three- and 24-h after intravenous administration of VSV at a dose of 1 × 10^9^ PFU. Neutrophils decreased from 37.4 ± 3.6% to 15.7 ± 3.3% of CD45^+^ cells in the bone marrow by 24-h (*p* < 0.0001; Figure 1A). Most of this decrease occurred after the three-hour timepoint, when 29.4 ± 4.5% remained in the bone marrow (*p* = 0.037 compared to controls that received PBS only). There was no significant change in the maturation status of neutrophils within the bone marrow in the first three hours after viral challenge (Figure 1B). However, by 24-h the proportion of mature neutrophils (defined as CD101^+^) in the bone marrow had significantly decreased to 19.5 ± 4.3% of all neutrophils compared to 41.5 ± 8.0% of all neutrophils in controls that received PBS only (*p* < 0.0001). Additional surface markers of interest were the CX chemokine receptor (CXCR)-4 and CXCR2. Both receptors are present on neutrophils, acting in an antagonistic manner to regulate neutrophil trafficking from the bone marrow. Ligation of CXCR4 provides a dominant retention signal to neutrophils in the bone marrow, while signaling through CXCR2 tends to promote egress into the blood stream. Interestingly, CXCR2^-^CXCR4^-^ neutrophils also emigrate from the bonne marrow [17]. The proportion of mature CXCR2^+^ (Figure 1C) and CXCR4^+^ (Figure 1D) neutrophils decreased in the bone marrow by 24-h post-VSV compared to PBS controls, in conjunction with an overall decrease in total neutrophils. CXCR2^+^ mature neutrophils decreased from 22.0 ± 7.0% to 5.0 ± 0.3% (*p* = 0.0025), while CXCR4^+^ mature neutrophils decreased from 13.6 ± 0.2% to 3.9 ± 0.3% (*p* = 0.0085). These results from the bone marrow indicate that within 24-h of intravenous administration of VSV, neutrophils likely emigrated from the bone marrow to traffic elsewhere in the body.

### 2.2. Neutrophils with Increased Expression of CXCR2 and Inflammatory Functions Appeared in the Bloodstream after Infection with VSV

**Figure 2:**
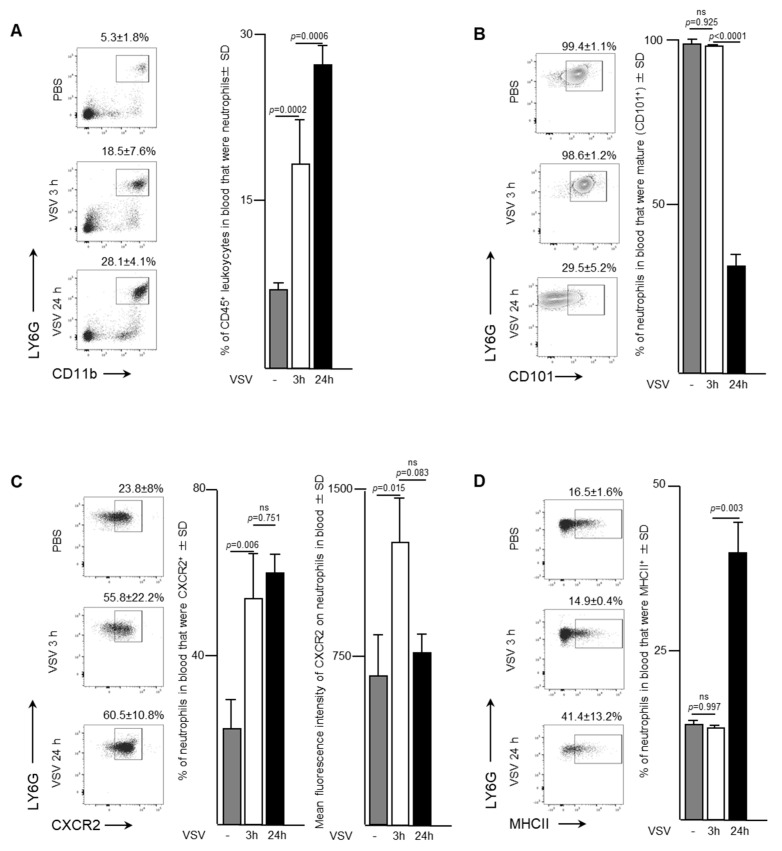
Neutrophils with increased surface density of CXCR2 and inflammatory functions entered the bloodstream in response to administration of vesicular stomatitis virus (VSV). Six- to eight-week-old female C57BL/6 mice were injected intravenously with 1 × 10^9^ plaque-forming units of VSV or phosphate-buffed saline (PBS) and euthanized three- or 24-h (h) later. Blood was assessed by flow cytometry to quantify neutrophils. Representative dot plots and graphs with means and standard deviations (SD) (*n* = 8/group) are shown for the proportion of (**A**) CD45^+^ leukocytes that were neutrophils (defined as CD45^+^Ly6G^hi^CD11b^hi^), (**B**) neutrophils that were mature (defined by the additional expression of CD101), (**C**) neutrophils expressing the lung-homing chemokine receptor CXCR2, and (**D**) neutrophils expressing the antigen presentation molecule major histocompatibility complex class II (MHCII). (ns = not significantly different).

To evaluate the trafficking pattern and phenotype of neutrophils emigrating from bone marrow, murine blood samples were evaluated three- and 24-h after a single dose of 1 × 10^9^ PFU of VSV administered intravenously. An increase in the proportion of neutrophils was apparent in the bloodstream within the first three-hours after mice were infected with VSV (Figure 2A). In fact, the proportion of neutrophils tripled from 5.3 ± 1.8% to 18.5 ± 7.6% (*p* = 0.0002) within the first three hours, followed by an additional increase to 28.1 ± 4.1% by 24-h (*p =* 0.0006). This increase was accompanied by a shift from a predominately mature population of CD101^+^ neutrophils within the blood three hours post-infection (98.5 ± 1.2% of total neutrophils) to an immature CD101^-^ subset (65.7 ± 10.9% of total neutrophils) (*p <* 0.0001; Figure 2B). These blood-derived neutrophils expressed significantly higher proportions of the chemokine receptor CXCR2 (Figure 2C). Control mice treated with PBS had 23.8 ± 8.0% of neutrophils expressing CXCR2 in their blood, which rapidly increased to 55.8 ± 2.2% by three hours (*p* < 0.0001), and then remained steady up to 24-h post-infection. The mean fluorescence intensity of CXCR2 expression on the surface of neutrophils was highest at the three-hour timepoint (1322 ± 163.6 MFI) compared to either the PBS-treated group (643.7 ± 206.9 MFI, *p* = 0.0146) or 24-h post-VSV (644 ± 314.6 MFI, *p* = 0.0825). The concentration of major histocompatibility complex class II (MHCII), an extracellular complex that presents antigens to CD4^+^ T cells after phagocytosis, did not change between zero- and three-hours post-infection (16.5 ± 1.6% vs. 14.9 ± 0.4%, *p* = 0.9966) (Figure 2D). However, a substantial increase in MHCII expression was observed by 24-h (41.4 ± 13.2%, *p* = 0.0028). These data correlated inversely with what was observed in the bone marrow, suggesting that neutrophils in VSV-infected mice rapidly emigrated into the bloodstream.

### 2.3. Neutrophils Were Mobilized from the Bone Marrow and Accumulated in the Lungs of Mice Following Intravenous Administration of VSV

**Figure 3:**
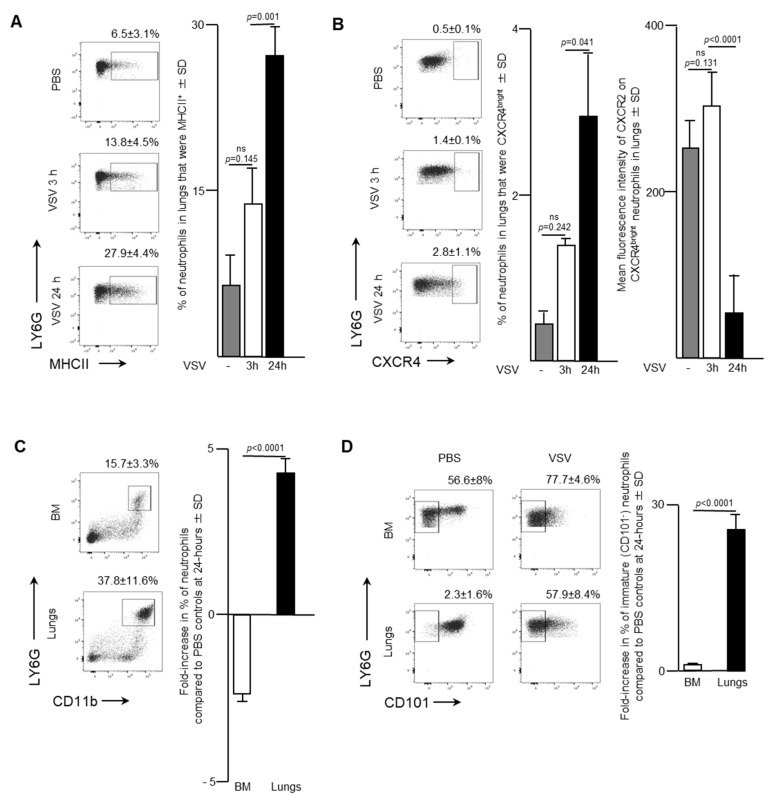
Neutrophils accumulated in the lungs of mice following intravenous administration of vesicular stomatitis virus (VSV). Six- to eight-week-old female C57BL/6 mice were injected intravenously with 1 × 10^9^ plaque-forming units of VSV or phosphate-buffed saline (PBS) and euthanized three- or 24-h (h) later. Femur-derived bone marrow (BM) and lungs that had been purged of blood were used to quantify neutrophils by flow cytometry. Representative dot plots and graphs with means and standard deviations (SD) (*n* = 8/group) are shown for the percentage of (**A**) pulmonary neutrophils (defined as CD45^+^Ly6G^hi^CD11b^hi^) that expressed major histocompatibility complex class II (MHCII), (**B**) pulmonary neutrophils that were CXCR4^bright^ (left graph) and the mean fluorescence intensity of CXCR2 on these CXCR4^bright^ neutrophils (right graph), and (**C**,**D**) the fold-change in the percentage of; (**C**) neutrophils; or (**D**) immature neutrophils (CD101^-^) in the BM and lungs compared to PBS-treated control mice. (ns = not significantly different).

Blood is a route by which neutrophils traffic into other solid tissues. Notably, lungs are known to be a common site by which activated leukocytes traffic. As such, the effect of administering a single intravenous dose of 1 × 10^9^ PFU of VSV on the pulmonary neutrophil population was also examined. Flow cytometry was used to analyze neutrophils in the lungs of infected mice. The proportion of neutrophils that expressed MHCII remained relatively unchanged three-hours post-VSV compared to control mice treated with PBS. However, there was a dramatic doubling in the percentage of pulmonary neutrophils that were MHCII^+^ from 13.8 ± 4.5% at three-hours post-infection to 24 h (27.9 ± 4.4%; *p* = 0.001; Figure 3A). This finding indicated that there was an increase in the innate antigen presentation capabilities of neutrophils in the lungs within 24-h after administration of VSV. Additionally, a significant increase in the proportion of neutrophils that were CXCR4^bright^ occurred 24-h after mice were treated with VSV (Figure 3B; 2.8 ± 1.1% versus 0.5 ± 0.1% for PBS-treated controls, *p* = 0.0406). This subset was monitored because it has been shown that CXCR4 signaling is involved with tumour metastasis [18]. Within the CXCR4^bright^ subset, a significant decrease in the mean fluorescent intensity of CXCR2 expression was observed to occur between three- and 24-h post-VSV administration (Figure 3B; 317.3 ± 26.6 MFI versus 69.8 ± 58.0 MFI, *p* < 0.0001). To highlight the overall magnitude of the shifts in neutrophil populations in the bone marrow and lungs, the fold-change in the proportion of neutrophils 24-h after administration of VSV was calculated relative to the same tissues in PBS-treated mice. An ~2.5-fold decrease in the proportion of neutrophils in the bone marrow correlated with an ~five-fold increase in the lungs (*p* < 0.0001; Figure 3C). Additionally, a 28-fold increase in the proportion of immature (CD101^-^) neutrophils in the lungs contrasted with an insignificant change in the bone marrow (*p* < 0.0001; Figure 3D). The sum total of the data suggested that infection with VSV caused a large proportion of neutrophils in the bone marrow to emigrate into the blood and then many of these blood-borne neutrophils trafficked into the lungs.

### 2.4. Upon Exposure to VSV a Significant Proportion of Splenic Neutrophils Expressed MHCII, the Inflammatory Cytokine IL-12, and Had Evidence of Having Acquired a Viral Transgene-Encoded Protein

**Figure 4:**
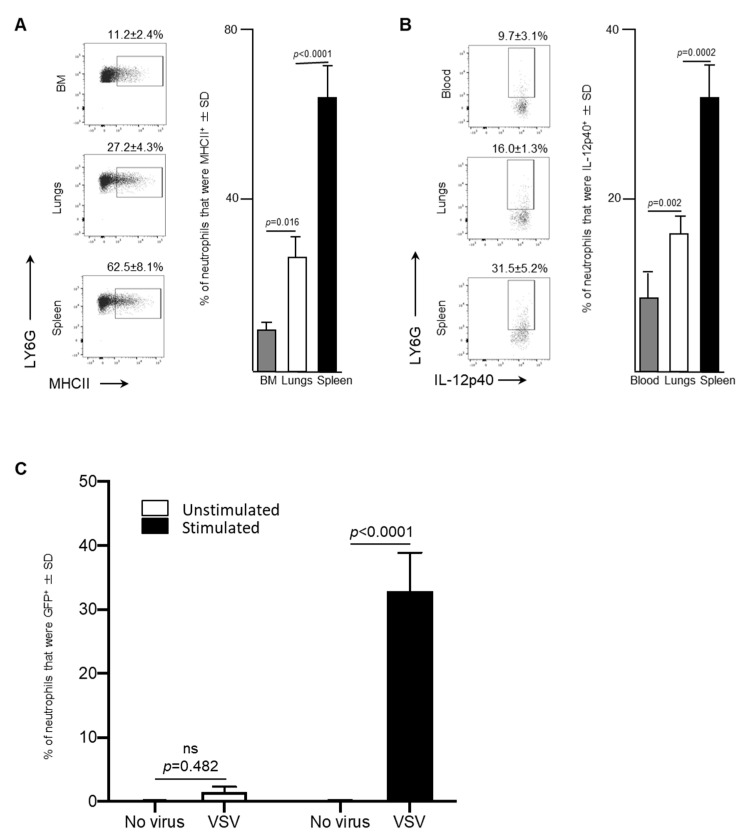
After exposure to vesicular stomatitis virus (VSV) splenic neutrophils expressed high concentrations of major histocompatibility complex II (MHCII), the inflammatory cytokine interleukin (IL)-12 and a viral transgene-derived protein. Six- to eight-week-old female C57BL/6 mice (*n* = 8/group) were injected intravenously with 1 × 10^9^ plaque-forming units of VSV or phosphate-buffed saline (PBS) and euthanized three- or 24-h (h) later. Bone marrow (BM)-, blood-, spleen- and lung-derived cells were used to quantify neutrophils by flow cytometry. Representative dot plots and graphs with means and standard deviations (SD) (*n* = 8/group) are shown for the proportion of (**A**) neutrophils (defined as CD45^+^Ly6G^hi^CD11b^hi^) that expressed MHCII in the BM, lungs and spleen, and (**B**) neutrophils in the blood, lungs and spleen that expressed IL-12p40. (**C**) To test neutrophils for intracellular expression of enhanced green fluorescent protein (GFP) encoded in the genome of VSV, bulk splenocytes were harvested from six- to eight-week-old naïve female C57BL/6 mice. Splenocytes were unstimulated or stimulated in vitro with anti-CD3 and anti-CD28 for four-hours and then left unexposed to the VSV or exposed to VSV-GFP at a multiplicity of infection of 10 for 18-h. Flow cytometry was used to determine the percentage of viable neutrophils (defined as CD45^+^Ly6G^hi^CD11b^hi^) that were GFP^+^; *n* = 10/group. (ns = not significantly different).

Leukocytes circulating in blood traffic through the spleen and, in the context of cancers, this organ can also serve as a reservoir of tumor-associated neutrophils [19]. Therefore, splenic neutrophils were also assessed in VSV-infected mice. A significant increase in the proportion of neutrophils that were MHCII^+^ in the spleen (62.5 ± 8.1%) was observed 24-h following administration of VSV, compared to the bone marrow (11.2 ± 2.4%, *p* < 0.0001) or lungs (27.2 ± 4.3%, *p* < 0.0001; Figure 4A). Moreover, IL-12p40, a cytokine that plays a major role in antigen presentation and is indispensable for clearance of viruses by regulating interferon-γ and other cytokines [20], was produced by a higher proportion of splenic neutrophils after 24-h (31.5 ± 5.2%) than the blood (9.7 ± 3.1%, *p* = 0.002) and lungs (16.0 ± 1.3%, *p* = 0.0002) (Figure 4B). This data demonstrated that the spleen had a larger proportion of neutrophils, with antigen presenting potential, compared to the bone marrow and lungs.

The antigen-presentation phenotype of splenic neutrophils led to the evaluation of the potential of these cells to acquire VSV-encoded gene products. To do this, an in vitro assay was conducted to determine the capacity of neutrophils in a bulk splenocyte culture to become GFP^+^ in the presence of media, containing CD3- and CD28-ligating antibodies. Eighteen-hours after exposure of splenocytes to VSV-GFP, neutrophils were assessed by flow cytometry to determine if they had acquired GFP (Figure 4C). A small proportion of neutrophils among the splenocytes exposed to VSV-GFP in the absence of anti-CD3/CD28 became GFP-positive (1.5 ± 0.8%) compared to the control cells that were not treated with VSV (0.07 ± 0.11%), but this was not significant (*p* = 0.482) (Figure 4C). However, when splenocytes were incubated in media containing anti-CD3 and anti-CD28, a significant proportion of the neutrophils within the culture had acquired the viral transgene GFP 18-h after in exposure to VSV-GFP (32.9 ± 5.9% versus 1.5 ± 0.8% control cells that had been pre-treated with anti-CD3/CD28 but were not exposed to VSV-GFP; *p* < 0.0001 (Figure 4C). This scenario was designed to recapitulate an environment in which activated T cells are present, similar to what is observed in most tumors [21]. In this setting, the neutrophils were able to acquire a non-structural protein encoded in the genome of VSV.

### 2.5. Administering VSV-GFP as Either a Single Dose or as a Second Dose 24-h after an Initial Treatment with VSV Significantly Increased the Proportion of GFP-Positive Neutrophils in the Spleen with a Concomitant Decrease of Total Neutrophil in Bone Marrow

**Figure 5:**
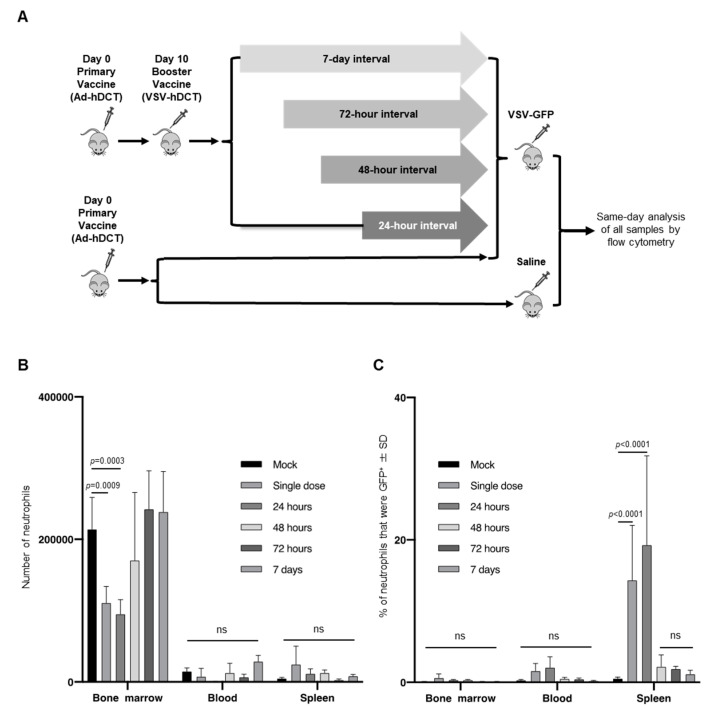
Dosing regimens with vesicular stomatitis virus (VSV) that used a single dose or two doses 24-h apart led to a significant increase in the proportion of splenic neutrophils expressing a viral transgene-encoded protein compared to two doses administered at 48-h or longer intervals. (**A**) In vivo experimental dosing design. Six- to eight-week-old female C57BL/6 mice were injected intramuscularly with 1 × 10^8^ infectious units of a replication-deficient human serotype 5 adenovirus vaccine with a transgene encoding the melanoma-associated antigen human dopachrome tautomerase (Ad-hDCT). Ten-days later mice received intravenous injections of 1 × 10^9^ plaque-forming units (PFU) of VSV-hDCT as a booster vaccine. Mice received a second intravenous dose of 1 × 10^9^ PFU of VSV-GFP at 24-, 48-, 72-h or 7-day intervals. Control mice received a single dose of VSV-GFP or PBS. Initiation of treatments were off-set so that all mice receiving VSV-GFP were treated on the same day. Femur-derived bone marrow, blood and spleens were harvested 18-h after treatment with VSV-GFP for flow cytometric analysis. (**B**,**C**) Bar graphs with means and standard deviations (SD) (*n* = 4/group) show (**B**) numbers of neutrophils (defined as CD45^+^Ly6G^hi^CD11b^hi^), and (**C**) the proportion of neutrophils that were GFP^+^. (ns = not significantly different).

Administering multiple doses of oncolytic viruses has been a common strategy to try to optimize treatment efficacy of virotherapies [22]. As such, an experiment was designed to determine the impact of intravenous administration of a single versus multiple doses of 1 × 10^9^ PFU of VSV on neutrophils (Figure 5A). To simulate a strategy [23] that is now in multiple human clinical trials, mice received a primary vaccine of 1 × 10^8^ infectious units (IU) of Ad-hDCT administered intramuscularly [23]. Ten-days later mice were boosted with VSV-hDCT. Mice subsequently received a second dose in the form of VSV-GFP at an interval of 24-, 48- or 72-h, or seven-days (Figure 5A). Control groups received Ad-hDCT followed by a single dose of VSV-GFP or Ad-hDCT followed by PBS.

There was an ~50% decrease in the relative number of neutrophils in the bone marrow 18-h after a single dose of VSV was administered (214,079 versus 110,906 compared to PBS-treated mice, *p* = 0.0009; Figure 5B), or if two doses were administered 24-h apart (95,144, *p* = 0.0003). This loss of neutrophils in the bone marrow was not evident where the interval between consecutive doses of VSV was greater than 24-h. Additionally, there was no significant change in the total number of neutrophils within the blood or spleen for any dosing protocol. 

The multi-dosing experimental design was also used to extend the previous in vitro experiment that monitored GFP^+^ neutrophils among bulk splenocytes stimulated with anti-CD3/CD28. Interestingly, a significant proportion of neutrophils in the spleen became GFP-positive 18-h after administering a single dose of VSV-GFP (14.3 ± 7.7%, *p* < 0.0001; Figure 5C) or after VSV-GFP was given to mice that had been vaccinated with VSV-hDCT 24-h prior (19.3 ± 12.5%; *p* < 0.0001) compared to sham-treated controls (<1%). There was no significant increase in the proportion of GFP-positive splenic neutrophils where the multi-dosing protocol used a 48-h or longer interval between doses. Nor did a significant proportion of neutrophils become GFP-positive in the bone marrow or blood. These data indicate that administering a single dose of VSV or two doses 24-h apart promoted the acquisition of a non-structural viral protein by splenic neutrophils, while extending the interval between doses beyond 24-h did not have this effect.

## 3. Discussion

The data presented here indicated that a rapid neutrophil response occurred within the bone marrow of female C57BL/6 mice after systemic administration of a single dose of 1 × 10^9^ PFU of VSV, which represents a typical route of delivery and dosage for cancer studies [23]. Specifically, the proportion of neutrophils in bone marrow had decreased substantially within 24-h post-infection (Figure 1). This coincided with increases in the proportions of neutrophils circulating in the blood and accumulating in the spleen and lungs (Figure 2, Figure 3 and Figure 4). This suggests the probability of sequential trafficking of neutrophils from the bone marrow to blood, and from the blood to the spleen, which filters the blood, and from the blood and/or spleen into the lungs. Neutrophils have been documented as entering the lungs in response to other viruses such as respiratory syncytial virus [24]. In the case of respiratory syncytial virus, this trafficking might predominantly be due to the pulmonary site of infection. In contrast, the VSV in this study was administered intravenously, suggesting that trafficking to the lungs might have largely been due to that tissue having a general tendency to attract leukocytes with an activated phenotype. With that said, infection of the lungs by VSV after intravenous delivery cannot be ruled out. Interestingly, splenic neutrophils exhibited a drastic upregulation of MHCII and inflammatory cytokines following administration of VSV. The spleen is an important secondary lymphoid tissue for coordinating immune responses, immune editing and antigen presentation [25], and thus, it is important to analyze it to determine the changes that oncolytic viruses might exert on the innate immune system.

The proportion of immature and mature neutrophils was examined, in this study, as the implications of maturity have recently been a subject of interest within the cancer community [26]. Between three- and 24-h post-infection with VSV, the majority of neutrophils in the bone marrow had adopted an immature phenotype, as indicated by their lack of expression of CD101 (Figure 1) [27]. This suggested that neutrophils emigrating from the bone marrow were adopting a progressively more immature phenotype over time, entering the blood stream and trafficking to the lungs, as has been observed previously for other infections [28]. Indeed, a majority of neutrophils in the blood and lungs had an immature phenotype 24-h post-infection (Figure 2 and Figure 3). Although neutrophil research, in relation to cancer, is in its infancy [26], the role of immature and mature neutrophil populations have had preliminary studies conducted. Immature neutrophils may have a role akin to myeloid-derived suppressor cells and mediate suppression and metastasis [26]. Understanding how the addition of an oncolytic virus changes circulating neutrophil populations may have wide applicability to the optimization of immunotherapies.

CXCR2 is chemokine receptor on neutrophils [29] that is crucial in regulating neutrophil homeostasis, and works in conjunction with CXCR4 to control neutrophil release from the bone marrow [17]. Mature CXCR2^bright^ neutrophils disappeared from bone marrow by 24-h after administration of VSV (Figure 1). In parallel, an increase in circulating neutrophils within the blood was observed (Figure 2). Gradually, by 24-h a high number of immature neutrophils (~70%) were detected circulating in the blood. Given the increase of neutrophils in the lungs (Figure 3) and spleen (Figure 4), these data based on chemokine receptors also suggests that administering VSV caused neutrophils to be released from the bone marrow and traffic to other tissues.

An unexpected observation was made with respect to neutrophils that expressed CXCR4, which is usually a retention marker. Specifically, it was demonstrated that upon infection with VSV, all the subsets of mature neutrophils, including those that were CXCR4^+^, decreased in the bone marrow (Figure 1). Studies have shown that granulocyte colony-stimulating factor (G-CSF), generated remotely at sites of inflammation, act systemically to stimulate murine neutrophil mobilization from the bone marrow [30]. Additionally, evidence suggests that neutrophil mobilization from the bone marrow by G-CSF [31] may be facilitated by the effects of G-CSF on CXCR4/CXCL12 signaling, by decreasing the concentration of CXCL12 in the bone marrow [32]. Knowing that virus-induced inflammation in animal models and humans is associated with an increase in serum levels of G-CSF [33], a decreased local concentration of CXCL12 in the bone marrow may have been why VSV caused the otherwise unexpected release of CXCR4^+^ neutrophils by 24-h post-infection. Interestingly, it has been shown that during acute inflammatory responses, aged neutrophils that are CXCR4^hi^ cease returning to the bone marrow and instead rapidly migrate to sites of inflammation [34]. Moreover, given that treatment of mice with G-CSF induces a decrease in the expression of CXCR4 on the surface of neutrophils [35], in conjunction with a reduction in CXCL12, this could be the basis for the VSV-induced emigration of mature neutrophils expressing CXCR4 from the bone marrow, as was shown here. However, this speculative mechanism of action would need to be confirmed in this model.

An increase in expression of MHCII on neutrophils was observed by 24-h post-infection within the blood, spleen and lungs compared to PBS-treated control mice. As time progressed, neutrophils upregulated this molecule, which is associated with antigen presentation. Further, splenic neutrophils progressively increased their production of IL-12p70 over time. This pro-inflammatory cytokine plays a critical role in modulating antigen presentation to lymphocytes. A growing body of evidence shows that neutrophils are capable of presenting antigens to CD4^+^ T cells [36]. Interestingly, 24-h after infection the spleen had a significantly larger proportion of MHCII^+^ neutrophils than either the bone marrow or lungs. This finding can be explained by the importance of the spleen during immune responses. Contact between antigen-specific T and/or B lymphocytes and antigen-presenting cells carrying corresponding antigens is increased in the spleen. The physical organization of the spleen allows it to facilitate low-probability interactions between antigen-presenting cells and cognate lymphocytes [25]. The results of this study demonstrated that splenic neutrophils upregulated MHCII upon exposure to VSV. This antigen-presentation potential of splenic neutrophils may regulate the T and B cell response to antigenic targets that OVs are engineered to express. Increasing evidence indicates that neutrophils can contribute to adaptive immunity by influencing antigen-specific responses. They can have an indirect effect on antigen presentation by activating dendritic cells [37] and they may even directly activate T cells by transporting and presenting antigens themselves [38,39,40,41].

Alongside the increase in splenic neutrophils with a pro-inflammatory phenotype, consisting of expression of high concentrations of MHCII and production of IL-12 (Figure 4), a small subset of CXCR4^bright^ neutrophils progressively appeared in the lungs (Figure 3). Since CXCR4 is associated with retention of cells in the bone marrow, this may have been a mechanism used to control tissue inflammation by supporting trafficking of pro-inflammatory neutrophils back to the bone marrow [42]. Indeed, this coincided with a downregulation of CXCR2, which was also consistent with restoration of a bone marrow-homing phenotype. Interestingly, senescent neutrophils in blood are known to upregulate expression of CXCR4, which allows them to return to the bone marrow for clearance from the body [43]. 

The ability of neutrophils to acquire non-structural proteins from VSV was explored in vitro using bulk splenocytes. The spleen was selected because it is the largest secondary lymphoid organ in the body, and as such, hosts a wide range of immunologic functions [25]. Neutrophils are established phagocytosing cells that can engulf microorganisms. After internalization, these invading organisms are destroyed via numerous mechanisms including reactive oxygen species and antimicrobial peptides. It has been previously demonstrated that neutrophils can engulf VSV or carry it on its cell surface [3]. Neutrophils are also capable of phagocytizing other viruses such as HSV [44] and internalizing viral proteins from cytomegalovirus after phagocytizing infected endothelial cells [45]. Intriguingly, we demonstrated that the presence of T cell-stimulating CD3- and CD28-ligating functional antibodies made a significant difference to whether neutrophils became GFP-positive after splenocytes were exposed to VSV-GFP. It is possible that the GFP^+^ neutrophils could stem from engulfing infected cells, phagocytosing the viral transgene-encoded protein after release from another cell type, or even from a direct productive infection of neutrophils. Indeed, other innate leukocytes such as dendritic cells have been shown to become directly and productively infected by VSV, ultimately causing these cells to die [46]. It is currently unknown whether this phenomenon in neutrophils would lead to viral clearance, promotion of presentation of VSV-encoded antigens, death of neutrophils or help disseminate the virus, and therefore, constitutes an important future area of research with potentially far-reaching implications for virotherapies.

An *in vivo* multi-dosing experiment, described here, allowed for exploration of the impact of VSV on neutrophil biology across a variety of dosing regimens that are common in clinical trials. A single dose of VSV or two doses administered 24-h apart resulted in a significant decrease in the number of neutrophils in the bone marrow. This effect was abrogated where the interval between consecutive doses of VSV was greater than 24-h. Notably, a single or 24-h-interval multi-dosing protocol with VSV resulted in approximately 20% of splenic neutrophils expressing GFP, which was used as a representative non-structural protein encoded as a transgene. This phenomenon was not readily apparent in either the blood or bone marrow. This data indicates that a single dose or multi-dosing with VSV at a 24-h interval has a different effect on the innate immune system than longer multi-dosing intervals. The results suggest that neutrophils may become resistant to the acquisition of a viral non-structural protein beyond 24-h of exposure to VSV. The underlying mechanism(s) is worthy of future investigation. These results suggest that optimization of dosing protocols should, therefore, include studying the effects on neutrophils and determining whether they have a net positive or negative effect before proceeding to clinical trials.

There are several limitations of this study that should be considered. The study lacks definitive proof of where neutrophils are trafficking. This could be addressed using an adoptive transfer experiment. The results described here demonstrated that there were significant changes to neutrophil sub-populations when an oncolytic virus is added, and that varying dosing protocols changed how these sub-populations responded. In a tumor-bearing host, intratumoral delivery of VSV would be expected to cause local inflammation and promotion of trafficking of neutrophils into the tumor microenvironment. However, it is not clear whether their accumulation in the tumor microenvironment would be beneficial as there are data supporting both interference and necessity of neutrophils in OV-mediated therapies [3]. Instead, it is likely that this question would have to be addressed on a model-to-model basis. Tumor-free mice were used in this study because the goal was to study the effect of OVs on neutrophils, without any other immunological perturbations, including the influence of an immunosuppressive tumor. Different cancer models have been shown to interact with the innate immune system in various and often contradictory ways. The number of neutrophils present in a tumor can have varying outcomes [47]. For example, neutrophilia has been associated with poor prognoses in renal carcinomas [48] and pancreatic adenocarcinomas [49], whereas neutrophils with cytotoxic effects can decrease lung metastasis [50]. Therefore, it is essential to characterize the general reaction of neutrophils to oncolytic viruses before the addition of other confounding variables. Understanding how a tumor influences trafficking and/or phenotypes of neutrophils would be a logical extension of this research.

Another future area of neutrophil research that should be explored is how other OVs influence neutrophil trafficking. Previous studies have demonstrated that there is no “one size fits all” approach to oncolytic virotherapy. Indeed, different OVs have unique properties that can make them more effective in a different array of tumor types. Determining how other OVs shift neutrophil trafficking, phenotypes and functions would expand our knowledge on the interactions between these critical innate leukocytes and cancer therapies.

## 4. Materials and Methods

### 4.1. Ethics and Biohazard Certification

Animal experimentation was approved under animal utilization protocol #3807 (8 October 2017) by the Animal Care Committee at University of Guelph, Guelph, Ontario, Canada. All experiments were conducted following the guidelines from the Canadian Council on Animal Care. Virus-related research was conducted in certified containment level-2 facilities under biohazard permit #A-367-04-19-05 (19 April 2005) issued by the University of Guelph’s biosafety committee.

### 4.2. Mice

Female C57BL/6 mice (strain code #027; Charles River Laboratories, Wilmington, MA, USA) ranging in age from five- to seven-weeks were housed in the animal isolation unit and the central animal facility at the University of Guelph under specific pathogen-free conditions with a 12-h light/dark cycle. They received food and water *ad libitum* and were accommodated to their environment for one-week prior to the initiation of experiments.

### 4.3. Viruses

The plasmid vector system for recombinant VSV (Indiana serotype; the parental virus from which the Δm51 variant had been derived [51]) was kindly provided by Dr. Brian Lichty, McMaster University, Hamilton, ON, Canada. The VSV was engineered to express the melanoma-associated antigen human dopachrome tautomerase (hDCT). A second VSV was engineered to express enhanced green fluorescent protein. VSV-GFP allowed flow cytometry-based assessment of intracellular viral transgene expression due to either infection or engulfment of infected cells. The VSVs were made using a previously described cloning method [51] and were cultured in Vero cells (ATCC, Manassas, VA, USA) that were confirmed to be mycoplasma-free and grown in Dulbecco’s modified Eagle’s medium (Thermo Fisher Scientific, Mississauga, ON, Canada) supplemented with 10% heat-inactivated bovine calf serum (Seradigm, Providence, UT, USA). VSVs were purified using sucrose gradient ultracentrifugation followed by dialysis in phosphate-buffered saline (PBS; HyClone, South Loga, UT, USA). The viruses were subsequently titered using a standard plaque assay with Vero cells.

A human serotype-5 adenovirus (Ad) that was replication deficient due to an E1/E3-deletion was used in a multi-dosing experiment to recapitulate a previously published prime-boost strategy [52] in mice. This virus was also kindly provided by Dr. Brian Lichty. The Ad was genetically engineered to express hDCT as described previously [12]. The virus was propagated in human embryonic kidney 293 cells (ATCC, Manassas, VA, USA) and purified using a cesium-chloride gradient. The non-oncolytic adenovirus was titered using an antibody that is specific to the adenoviral hexon protein (Abcam, Cambridge, UK; catalogue #ab8249).

### 4.4. Tissue Processing

Retro-orbital blood, lungs, spleens and bone marrow were removed from mice to conduct flow cytometry analyses. Blood was collected in microtubes containing 10 µL of heparin (at a concentration of three µg of heparin/mL of Hank’s Balanced Salt Solution (HBSS; Hyclone) to prevent clotting. The vasculature of the lungs was perfused with HBSS to purge blood. Lungs were then minced, digested with collagenase IV and DNase (Thermo Fisher Scientific) for 30 min at 37 °C and then passed through a strainer with 70 µm pores to obtain a single-cell suspension. Dissected spleens were cut in half and pressed between frosted ends of two sterile glass microscope slides to release cells. Bone marrow was flushed from the diaphyses of dissected femurs with HBSS using an 18-gauge needle. Epiphyses were crushed in HBSS and then filtered through a strainer with 70 µm pores. These cells were combined with those obtained from the diaphyses. Tissue-derived cells were treated with ammonium-chloride-potassium lysis buffer to remove erythrocytes. The cells were then enumerated using an improved Neubauer counting chamber and equal numbers of cells from each mouse were assessed by flow cytometry.

### 4.5. Staining for Analysis by Flow Cytometry

Five antibody-based staining panels were designed to assess a variety of surface markers and cytokines pertinent to neutrophil biology (Supplementary Table S1). The cells were first incubated with anti-CD16/CD32 (clone 93; BioLegend, San Diego, CA, USA) at 4 °C for 15 min to prevent antibodies from binding non-specifically to Fc receptors. The wells were then centrifuged at 500× *g* for five minutes and rinsed with PBS and incubated with surface staining antibodies at 4 °C for 20 min. Zombie aqua™ fixable viability dye (BioLegend, San Diego, CA, USA) or 7- aminoactinomycin D (7AAD; BioLegend, San Diego, CA, USA) was included to exclude dead cells from the analyses. The stained cells were then suspended in PBS containing 0.5% bovine serum albumin and immediately analyzed on a FACS Canto II flow cytometer with data acquired using FACSDiva 8.0.1 software (BD Biosciences, Mississauga, ON, Canada). Data were analyzed using FlowJo v.10.1 software (FlowJo LLC, Ashland, OR, USA). Exported data were graphed and statistically analyzed using GraphPad Prism version 8 software (GraphPad, San Diego, CA, USA).

For detection of intracellular cytokines, cells were non-specifically stimulated with phorbol 12-myristate 13-acetate (10 ng/mL, Sigma-Aldrich, St. Louis, MO, USA) and ionomycin (500 ng/mL, from *Streptomyces conglobatus*, Sigma-Aldrich, St. Louis, MO, USA) for four hours at 37 °C. Subsequently, brefeldin-A (BioLegend, San Diego, CA, USA) was added for eight-hours to inhibit protein export by the Golgi apparatus. After the incubation period, staining of surface markers proceeded as described above. After staining with a viability dye, the samples were fixed using fixation buffer (BioLegend, San Diego, CA, USA) and permeabilized using intracellular staining perm-wash buffer (BioLegend, San Diego, CA, USA) at 4 °C for 30 min and then stained with cytokine-specific antibodies at 4 °C for 20 min.

### 4.6. Gating Strategy

Gating was conducted using FlowJo software v.10.1 (Supplementary Figure S1). Doublet cells were excluded from analyses using forward scatter (FSC)-area versus FSC-width and side scatter (SSC)-area versus SSC-width dot plots. Non-viable cells were excluded, and leukocytes were selected when panel space allowed by using the pan-leukocyte marker CD45. The neutrophils were defined as CD45^+^CD11b^hi^LY6G^hi^ [53]. 

### 4.7. In Vitro Splenic Neutrophil Study Design

To determine if neutrophils could acquire viral transgenes after splenocytes were exposed to VSV, an in vitro experiment was conducted. A 96-well plate was coated with 10 µg/mL of anti-CD3 (BioLegend, San Diego, CA, USA, Clone 145-2C11) overnight at 4 °C. Wells were washed three times with 200 µL of PBS. Spleens were processed as previously described from naïve female C57BL/6 mice (Section 2.4) and incubated in 2 µg/mL of anti-CD28 (BioLegend, San Diego, CA, USA, Clone 37.51) for four-hours at 37 °C. After the incubation period the wells were washed, and cells were incubated with VSV-GFP overnight for 18-h at 37 °C. The cells then underwent surface staining (panel #5 in Supplementary Table S1) and were immediately analyzed on a FACS Canto II flow cytometer to identify GFP^+^ neutrophils in stimulated versus unstimulated experimental groups.

### 4.8. In Vivo Multi-Dosing Study Design

To determine how single- versus multi-dosing with oncolytic viruses might affect neutrophil populations, an in vivo experiment was designed. Mice received a primary intramuscular immunization with 1 × 10^8^ infectious units (IU) of non-oncolytic Ad-hDCT. Ten-days later, mice received an intravenous booster vaccine of 1 × 10^9^ plaque forming units (PFU) of VSV-hDCT as had been described previously [12]. Mice then received a second dose of VSV-GFP at seven-, three-, two- or one-day intervals (Figure 5A). Mice that had only received a single dose of VSV-GFP or PBS ten days after the Ad vaccine were included as controls. Bone marrow-, blood-, and spleen-derived cells from these mice were assessed by flow cytometry eighteen-hours after the final dose of VSV-GFP was administered. This analysis included assessment of expression of GFP in cells.

### 4.9. Statistical Analysis

All statistical analyses were conducted using GraphPad Prism version 8 software. One-way analysis of variance with Tukey’s multiple comparisons test was used when there was one variable with more than two groups (Figure 1 and Figure 2). Student’s *t*-tests were used when there was one variable with two groups (Figure 3 and Figure 4). Two-way analysis of variance with Tukey’s multiple comparisons test was used when there were two variables (Figure 5). Differences were deemed significant for *p*-values ≤0.05. Graphs show means and standard deviations of the means. The mean fluorescence intensity (MFI) of surface receptors was measured by calculating the geometric mean within the fluorochrome channel of interest using FlowJo software, Version 10. Ashland, OR, USA.

## 5. Conclusions

Neutrophils are the first responders to sites of inflammation and infection. These innate cells play complex roles in viral infections and cancers. Expanding our knowledge of how neutrophil populations shift in response to the addition of an oncolytic virus, such as VSV is important to understand how oncolytic virotherapies interact with the immune system and to improve virus-based immunotherapeutics. This study established that administering VSV resulted in a rapid and dramatic change in the systemic distribution and phenotype of neutrophils. Specifically, neutrophils appeared to emigrate from the bone marrow, into the blood, and spleen and ultimately accumulate in the lungs. They adopted a progressively more immature phenotype over time. Splenic neutrophils upregulated MHCII and expressed IL-12, thereby, gaining antigen-presentation potential. This correlated with the adoption of a bone-marrow re-homing phenotype for neutrophils in the lungs, possibly in an attempt to reduce inflammation in this sensitive organ. Changing the dosing strategy resulted in various outcomes to neutrophils, including impacting their ability to acquire a non-structural viral protein within the spleen. These results provide a solid foundation of methods and results that researchers can leverage when designing and optimizing novel viral immunotherapies.

## Supplementary Materials

Supplementary materials can be found at https://www.mdpi.com/1422-0067/21/17/6347/s1.

## Abbreviations

Ad	E1/E3-deleted replication-deficient human serotype 5 adenovirus
7-AAD	7-aminoactinomycin D
CXCR	CX chemokine receptor
FSC	forward scatter
GFP	enhanced green fluorescent protein
G-CSF	granulocyte colony-stimulating factor
HBSS	Hank’s Balanced Salt Solution
hDCT	human dopachrome tautomerase
IU	infectious units
IL	interleukin
MHCII	major histocompatibility complex class II
MFI	mean fluorescence intensity
MyD88	myeloid differentiation factor 88
OVs	oncolytic viruses
PBS	phosphate-buffered saline
PFU	plaque-forming units
SSC	side scatter
VSV	replication-competent vesicular stomatitis virus, Indiana strain
